# The Pulmonary Extracellular Matrix Is a Bactericidal Barrier Against *Haemophilus influenzae* in Chronic Obstructive Pulmonary Disease (COPD): Implications for an *in vivo* Innate Host Defense Function of Collagen VI

**DOI:** 10.3389/fimmu.2018.01988

**Published:** 2018-08-31

**Authors:** Suado M. Abdillahi, Ramesh Tati, Sara L. Nordin, Maria Baumgarten, Oskar Hallgren, Leif Bjermer, Jonas Erjefält, Gunilla Westergren-Thorsson, Birendra Singh, Kristian Riesbeck, Matthias Mörgelin

**Affiliations:** ^1^Infection Medicine, Department of Clinical Sciences, Lund University, Lund, Sweden; ^2^Respiratory Medicine and Allergology, Department of Clinical Sciences, Lund University, Lund, Sweden; ^3^Airway Inflammation and Immunology, Department of Experimental Medical Science, Lund University, Lund, Sweden; ^4^Lung Biology, Department of Experimental Medical Science, Lund University, Lund, Sweden; ^5^Clinical Microbiology, Department of Translational Medicine, Lund University, Malmö, Sweden; ^6^Colzyx AB, Medicon Village, Lund, Sweden

**Keywords:** antimicrobial activity, bronchopulmonary infection, collagen VI, COPD, extracellular matrix, *Haemophilus influenzae*, innate immunity, pulmonary fibrosis

## Abstract

Non-typeable *Haemophilus influenzae* (NTHi) is a Gram-negative human commensal commonly residing in the nasopharynx of preschool children. It occasionally causes upper respiratory tract infection such as acute otitis media, but can also spread to the lower respiratory tract causing bronchitis and pneumonia. There is increasing recognition that NTHi has an important role in chronic lower respiratory tract inflammation, particularly in persistent infection in patients suffering from chronic obstructive pulmonary disease (COPD). Here, we set out to assess the innate protective effects of collagen VI, a ubiquitous extracellular matrix component, against NTHi infection *in vivo. In vitro*, collagen VI rapidly kills bacteria through pore formation and membrane rupture, followed by exudation of intracellular content. This effect is mediated by specific binding of the von Willebrand A (VWA) domains of collagen VI to the NTHi surface adhesins protein E (PE) and *Haemophilus* autotransporter protein (Hap). Similar observations were made *in vivo* specimens from murine airways and COPD patient biopsies. NTHi bacteria adhered to collagen fibrils in the airway mucosa and were rapidly killed by membrane destabilization. The significance in host-pathogen interplay of one of these molecules, PE, was highlighted by the observation that it confers partial protection from bacterial killing. Bacteria lacking PE were more prone to antimicrobial activity than NTHi expressing PE. Altogether the data shed new light on the carefully orchestrated molecular events of the host-pathogen interplay in COPD and emphasize the importance of the extracellular matrix as a novel branch of innate host defense.

## Introduction

*Haemophilus influenzae* is a Gram-negative microorganism and an exclusively human commensal and pathogen. It colonizes the nasopharyngeal mucosa of most healthy pre-school children at a very early age (1–4). Based on the presence or absence of a polysaccharide capsule, this microorganism is divided into encapsulated, that is, typeable strains (serotypes a-f) and unencapsulated strains (non-typeable *Haemophilus influenzae*, NTHi). NTHi predominantly causes disease in the respiratory tract, but can also invade the blood stream [refs in (5–7)]. Although this organism is normally a commensal, its pathogenic properties as well as defects in host defense by underlying medical conditions, such as immunodeficiency, chronic lung disease, or acute viral infection, may lead to development of infection. The pathogen may spread from the upper airways to the lower respiratory tract. It causes infections like otitis media, sinusitis, conjunctivitis, bronchitis, pneumonia, and secondary chronic respiratory disease. NTHi also plays an increasingly important role in the pathogenesis of chronic lower respiratory tract inflammation (8). It is frequently isolated in individuals with COPD (chronic obstructive pulmonary disease), both during stable disease and acute exacerbations (6, 7).

In response to host defense mechanisms, NTHi has evolved certain immune evasion strategies such as the production of immunoglobulin A (IgA) protease, circumvention of complement-mediated attacks by accumulation of complement inhibitors, or binding to immunoglobulins (9–12). Other mechanisms, which often are implicated as immune evasion strategies, are the ability to hide intracellularly or to invade into the local extracellular tissue compartment. For this purpose, NTHi has evolved the ability to interact with extracellular matrix components such as collagens I, III, IV, and V, laminin, vitronectin, and fibronectin (13–20). When the tightly sealed epithelial cell barrier is compromised due to mechanical and chemical trauma and/or viral infections, extracellular matrix components are exposed as adhesive targets for bacterial adhesins. In addition, there is emerging evidence for a dual role of extracellular matrix molecules by conferring innate host defense through specific domains with antimicrobial properties (21–24). Thus the extracellular matrix in the bronchopulmonary system, but also in connective tissues in general, may act like fly paper by both attracting, containing and rapidly killing pathogen intruders.

The interplay between NTHi and extracellular host targets is transmitted by a spectrum of bacterial surface factors. Examples are the recently described NTHi protein E (PE), a 16-kDa outer membrane lipoprotein that functions as an adhesin in interactions with host epithelial cells and in subversion of the host innate immune response (25, 26). It induces a proinflammatory epithelial cell response leading to IL-8 secretion and upregulation of ICAM-1 (CD54). PE binds to vitronectin and laminin which leads to reduced MAC-induced hemolysis and contributes to bacterial serum resistance (15, 27). *Haemophilus* adhesion and penetration protein (Hap) is an autotransporter and adhesin that binds fibronectin, laminin, and collagen IV (15, 18). Hap is ubiquitous among NTHi isolates and mediates adhesion to respiratory epithelial cells, invasion, and bacterial aggregation. Presumably Hap facilitates NTHi colonization by promoting microcolony formation on respiratory epithelial cells (28, 29). Hia, or *Haemophilus influenzae* adhesin, is a trimeric autotransporter and adhesin that promotes NTHi adherence to epithelial cells (30, 31). Like PE and Hap, it is a virulence factor that promotes NTHi infection. It is likely that these different virulence factors work together to facilitate adherence to the respiratory epithelium, microcolony and biofilm formation, immune evasion, and thus persistent infection of the host.

Collagen VI is a remarkable multidomain molecule present in the extracellular matrix of virtually all connective tissues. It is a heterotrimer of different alpha chains, which are encoded by six different genes (*COL6A1, COL6A2, COL6A3, COL6A4, COL6A5*, and *COL6A6*). The molecule has a complex multi-step pathway of biosynthesis and assembly, both intra- and extracellularly, leading to a characteristic extracellular network of beaded microfibrils. During recent years, it has become increasingly evident that collagen VI exerts several key roles in a wide range of tissues, both in physiological and pathological conditions (32). These range from unique biomechanical roles, characteristic for the collagen family, to a range of cytoprotective functions such as counteracting apoptosis and oxidative damage, regulating autophagy and cell differentiation and maintenance of cell integrity. In previous work, collagen VI was shown to be upregulated in the airway walls during tissue remodeling in pulmonary fibrosis (24, 33). We recently identified this collagen as a target for microorganisms, exerting both adhesive and antimicrobial properties, identifying this molecule as a key player in connective tissue innate immunity (24, 34).

However, important aspects of the interaction between NTHi and the bronchopulmonary system in chronic airway infections and COPD are still not well defined. Therefore, in this study, we set out to assess the innate protective effects of collagen VI in the airway mucosa against NTHi infection *in vivo*. In regions of epithelial denudation in COPD, the lamina propria becomes exposed and accessible to intruding pathogens. In this environment adhesive and antimicrobial properties of collagen VI networks in the tissue are beneficial for the host. Indeed, collagen VI significantly kills NTHi at physiological pH and ionic strength through membrane perforation, destabilization and subsequent exudation of cytoplasmic content. This effect is mediated by specific binding of the NTHi surface factors PE and Hap to the von Willebrand factor A (VWA) domains of collagen VI. Thus, in particular, our findings identify PE and Hap as novel collagen VI adhesins. Interestingly, PE protects NTHi against the antimicrobial properties of this collagen in the pulmonary extracellular matrix. Similar observations were made in murine airway specimens and in biopsy samples obtained from COPD patients. These findings emphasize the *in vivo* importance of the extracellular matrix in innate immunity against NTHi, one of the most common Gram-negative respiratory pathogens. Furthermore, they shed new light on some basic prerequisites for the intimate and carefully fine-tuned relationship between pathogens and the human host.

## Materials and methods

### Bacterial strains and growth conditions

Non-typeable *Haemophilus influenzae* strain 3655 was cultured on chocolate agar plates or in brain heart infusion (BHI, Sigma-Aldrich Sweden, Stockholm, Sweden) liquid broth supplemented with NAD and hemin (both at 10 μg/ml, Sigma-Aldrich). Manufacture of isogenic NTHi 3655 mutant strains and *E. coli* expressing adhesins was used as described in (14). NTHi3655Δ*pe* was cultured in BHI supplemented with 17 μg/mL kanamycin (Merck, Darmstadt, Germany), and NTHi3655Δ*hap* was incubated with 3 μg/mL chloramphenicol (Sigma-Aldrich). Both kanamycin and chloramphenicol were used for growth of the NTHi3655Δ*pe/*Δ*hap*. Bacteria were grown to 2 × 10^9^ cfu/ml at 37°C in a humid atmosphere containing 5% CO_2_.

### Antibacterial activity assay

Bacteria were grown to the mid logarithmic phase as described above (OD_620_ ≈ 0.4), harvested by centrifugation at 1000 *x g* for 10 min, and washed once in TBS buffer [150 mM NaCl, 50 mM Tris-HCl, pH 7.4, supplemented with 0.8 mM MgSO_4_ modified from (35)]. Bacterial suspensions were adjusted to 2 × 10^9^ colony-forming units (cfu) per ml. The bacteria were further diluted in TBS and incubated with 3 μM LL-37 (Innovagen, Lund, Sweden) or 2 μM collagen VI for different times (0, 30, 60, and 120 min, respectively). In another setting, bacteria were incuabted for 2 h with LL-37 (10 nM, 100 nM, 1 μM and 2 μM) or collagen VI (10 nM, 100 nM, 1 μM and 2 μM). Bacteria incubated with TBS were used as controls. Samples were incubated at 37°C in a humid atmosphere containing 5% CO_2_. Serial dilutions were plated on chocolate agar plates and incubated overnight at 37°C, 5% CO_2_, and the number of cfu was thereafter determined by counting visible colonies. All experiments were performed in triplicate.

### Reagents, labeling of proteins, and binding assays

Native, intact full-length collagen type VI microfibrils were extracted from bovine cornea by collagenase digestion (36) with modifications as described in (24, 34). Antibodies (1014+) were a kind gift of Dr. Rupert Timpl [MPI, Martinsried, Germany, for refs see (37, 38)]. PE, Hap, and Hia were purified and characterized as previously described in (25, 27). Proteins were radiolabeled with ^125^I using iodobeads (Pierce, Rockford, IL, USA) according to the manufacturer's specifications. Binding of radiolabeled protein to bacteria was performed as described in (34). For electron microscopy, antibodies were labeled with colloidal gold as described earlier (39). Bacteria were grown to mid-logarithmic phase, washed, and diluted to 2 × 10^9^ cfu/ml in TBS supplemented with 5 mM glucose (TG-buffer) and then incubated with gold-labeled proteins for 1 h at 37°C. Alternatively, collagen VI microfibrils were incubated with equimolar amounts of gold conjugates of PE, Hap, Hia (both at 5 nm), and collagen VI antibodies (10 nm), respectively, under similar conditions. Samples were prepared for negative staining as described below.

### Human lung tissues

The present study involved 11 subjects with moderate to severe COPD (GOLD stage II–IV). Lung tissue was obtained in association with lung lobectomy due to suspected lung cancer, a routine procedure to collect tissues from COPD patients. Only patients with solid tumors with visible borders were included in the study, and tissue was obtained as far from the tumor as possible. For histological sample preparation, care was taken to immerse the tissue in 4% paraformaldehyde immediately after surgical excision.

### Human pulmonary fibroblasts and cell culture conditions

Fibroblasts were isolated from biopsies from control subjects and from tissue sample explants obtained from COPD patients as described in detail in (24). Briefly, small pieces of biopsies from healthy individuals and COPD patients (GOLD stage IV) were allowed to adhere to cell culture flasks for 4 h and kept in cell culture medium at 37°C until outgrowth of fibroblasts was observed. All experiments were performed at passages 3–6. The cell cultures were continuously stained with antibodies against vimentin and prolyl-4 hydroxylase to verify the mesenchymal identity and to estimate the purity.

### Adhesion of NTHi to human lung biopsies

Paraffin embedded human lung tissue specimens were sectioned and mounted on glass slides according to routine protocols. For bacterial adhesion experiments, they were deparaffinized with tissue clear and rehydrated through a descending series of ethanol (100 to 50%). The specimens were inoculated with 2 × 10^9^ cfu/ml of NTHi in PBST (PBS containing 0.05% Tween 20) for 1 h at 37°C in a wet chamber. Unbound bacteria were removed by extensive washing with PBST. Tissue sections were then fixed with 2.5% glutaraldehyde in cacodylate buffer (0.15 M sodium cacodylate, pH 7.4) overnight at room temperature. Specimens were prepared for scanning electron microscopy as described below.

### Adhesion of NTHi to human pulmonary fibroblasts

Fibroblasts from healthy and COPD lung tissues were incubated with 2 × 10^9^ cfu/ml of bacteria for 1 h at 37°C in a humid atmosphere with 5% CO_2_. After a washing step, cells were fixed with 4% formaldehyde and 2.5% glutaraldehyde in PBS for 2 h at room temperature. In some experiments, for pre-embedding immunogold labeling of collagen VI in matrix fibrils, sections were incubated with a 1:100 dilution of anti-collagen VI antibody and protein A/G–coated colloidal gold particles (10 nm; British BioCell International, Cardiff, UK). After washing, samples were fixed with 4% formaldehyde and 2.5% glutaraldehyde. Specimens were subsequently prepared for scanning electron microscopy as described below.

### Animals

Six- to eight-week-old female C57BL/6 mice were supplied by Jackson Laboratories (Bar Harbor, ME). They were housed under standard conditions and provided with standard rodent chow and water *ad libitum*. All mice were housed with a 12 h light-dark cycle, under specific pathogen-free conditions. Animals were exposed to 12 3R4F reference cigarettes (Tobacco and Health Research Institute, University of Kentucky, Lexington, KY, USA) with the filters removed in a SIU-48 whole-body cigarette smoking machine (Promech Lab, Vintrie, Sweden). Mice were exposed twice daily for 50 min, on 5 consecutive days for 24 weeks. Control mice were exposed to room air only.

### Intratracheal challenge with NTHi

Mice (*n* = 3 per group) were anesthetized to a surgical plane using isoflurane. The trachea was then surgically exposed and bacteria were instilled intratracheally using a 29-gauge needle. The wound was closed using 5.0 silk sutures, and the animals were allowed to recover. They were then returned to normal housing with food and water *ad libitum*. After 30 and 120 min, respectively, the animals were sacrificed with CO_2_ and the lungs were collected for analysis. For determination of viable bacteria lung pieces were washed with PBS and homogenized in a volume of 400 μl of PBS using a Multi-Gen 7 homogenizer (Pro Scientific, Monroe, CT, USA). The samples were vortexed, diluted, and plated in triplicate on chocolate agar plates. The plates were incubated overnight at 37°C, and the number of cfu was counted. For determination of contaminating normal flora (microbiome) one group of mice was injected with PBS alone and treated as above. In parallel, lung tissue samples were prepared for scanning electron microscopy as described below.

### Electron microscopy and immunohistochemistry

For negative staining and transmission electron microscopy, samples were adsorbed to 400 mesh carbon-coated copper grids and stained with 0.75% (w/v) uranyl formate as recently described in detail (40). Transmission immunoelectron microscopy was performed as described earlier (41). In short, specimens were fixed in 150 mM sodium cacodylate, 2.5% glutaraldehyde, pH 7.4 and embedded in Epon. Ultrathin sections were subjected to antigen retrieval with sodium metaperiodate and subsequently incubated with primary antibodies, followed by detection with species-specific secondary antibody-gold conjugates. Samples were examined in a Philips/FEI CM 100 TWIN transmission electron microscope (FEI Co, Hillsboro, OR, USA) at 60-kV accelerating voltage. Images were recorded with a side-mounted Olympus Veleta camera with a resolution of 2,048 × 2,048 pixels (2k × 2K) using ITEM^TM^ software. For scanning electron microscopy, specimens were fixed over night at RT with 2.5% glutaraldehyde in cacodylate buffer. They were then washed with cacodylate buffer and dehydrated with an ascending ethanol series from 50% (v/v) to absolute ethanol. The specimens were thereafter subjected to critical point drying with carbon dioxide and absolute ethanol was used as an intermediate solvent. The tissue samples were mounted on aluminum holders, sputtered with 20 nm palladium/gold, and examined in a Philips/FEI XL 30 FESEM scanning electron microscope using an Everhart-Tornley secondary electron detector. Image processing was done with the Scandium software for simple image acquiring and auto-storage into the Scandium database. All electron microscopic work was performed at the Core Facility for Integrated Microscopy, Panum Institute, University of Copenhagen (Denmark). Contrast, brightness, and pseudocolours were adjusted in Adobe Photoshop CS6.

### Ethics statement

Adult patients (*n* = *7*) suffering from very severe COPD (spirometric GOLD stage IV) who were undergoing lung transplantation at Lund University Hospital 2006–2008 were included in this study. All subjects gave their written informed consent to participate in the study, which was approved by the local ethics committee in Lund, Sweden (LU339-00). All *in vivo* procedures were carried out under local ethics approval and in strict accordance to the 1986 Animals (Scientific Procedures) Act. Experiments were approved by the Animal Research Ethics Board at Lund University and conducted in accordance with the ethical guidelines outlined by the Swedish Council on Animal Care (no. FEK 213/2005, FEK 91/2006, and FEK 413/2008).

### Statistical analysis

Evaluation of the data was performed by counting particles in at least 50 different cellular profiles from three different experiments each. Statistical analysis was performed using GraphPad Prism, Version 7.00. The *p* value was determined by using the unpaired *t*-test (comparison of two groups) and the threshold used for significance was 0.05. All experiments were performed at least three times, if not otherwise mentioned.

## Results

### Kinetics of collagen VI-induced NTHi killing by membrane permeabilization

**Figure 1:**
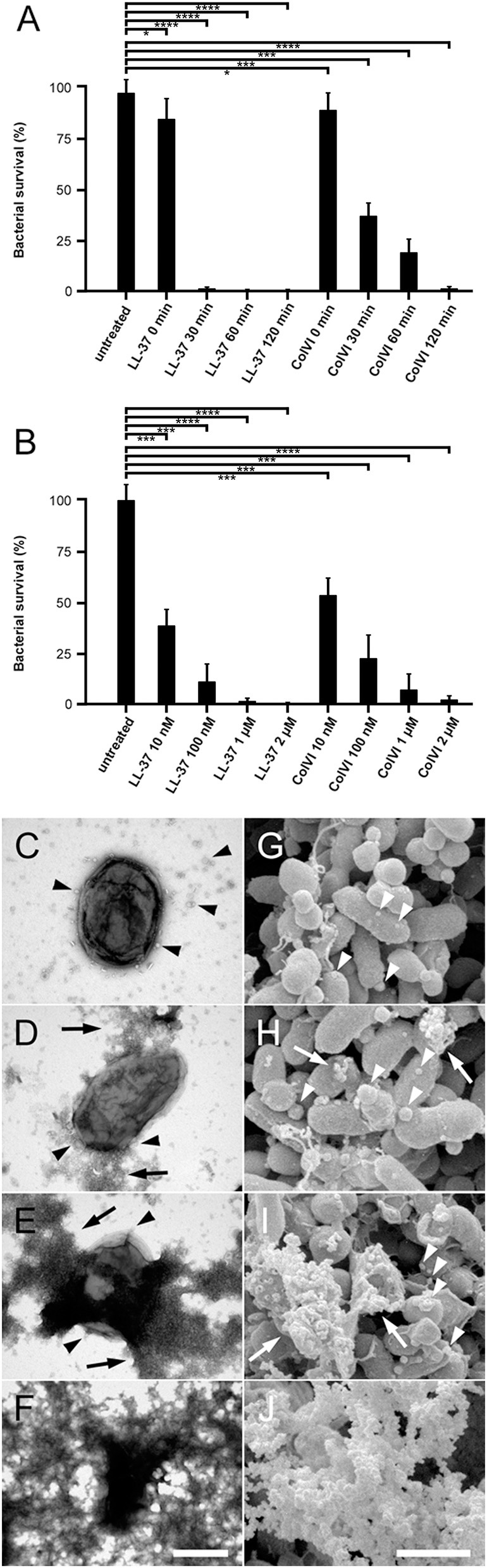
Collagen VI kills NTHi by membrane permeabilization and rupture. **(A)** Bacteria were incubated with 2 μM collagen VI for 0, 30, 60, and 120 min at 37°C, respectively. **(B)** Bacteria were treated with different molar quantities of LL-37 or collagen VI between 10 nM and 2 μM, as indicated, for 2 h at 37°C, respectively. Numbers of bacterial colonies after incubation with collagen VI were determined in viable count assays. Incubation in buffer alone (untreated) or LL-37 served as negative and positive controls, respectively. The dots represent individual experiments with the respective strains. Values are expressed as percent bacterial survival. **(C–F)**, negative staining and transmission electron microscopy, **(G–J)** scanning electron microscopy of NTHi killing. Incubation with collagen VI leads to membrane blebbing (arrowheads) and exudation of cytoplasmic contents (arrows). Finally, large scale membrane destabilization severely impairs NTHi architecture **(F, J)**. The scale bars represent 500 nm **(C–F)** and 1 μm **(G–J)**.

For a comprehensive analysis of early and late events during collagen VI-induced killing, NTHi bacteria were incubated with dilute solutions of this molecule in physiological saline for different time points (Figure 1). Collagen VI was compared to the “classical” cathelicidin peptide LL-37 at similar concentrations. The bactericidal effect was assessed by viable count assays (Figures 1A,B) and visualized by transmission electron microscopy (Figures 1C–F) and high resolution scanning electron microscopy (Figures 1G–J). Already after 30 min exposure to collagen VI bacterial survival was significantly reduced. After 2 h the microorganisms were cleared by both collagen VI and LL37 and no more colonies were present in viable count assays (Figure 1A). Collagen VI killed NTHi almost as efficiently as LL-37. Electron microscopic inspection of the different time points revealed that collagen VI rapidly induced membrane blebbing (arrowheads) and exudation of cytoplasmic content (arrows) within the first 30 min. After 2 h overall bacterial architecture was severely compromised by large-scale membrane rupture and intact bacteria were barely observed (Figures 1F,J).

### Pore formation is an early event during collagen VI-induced NTHi membrane rupture

**Figure 2:**
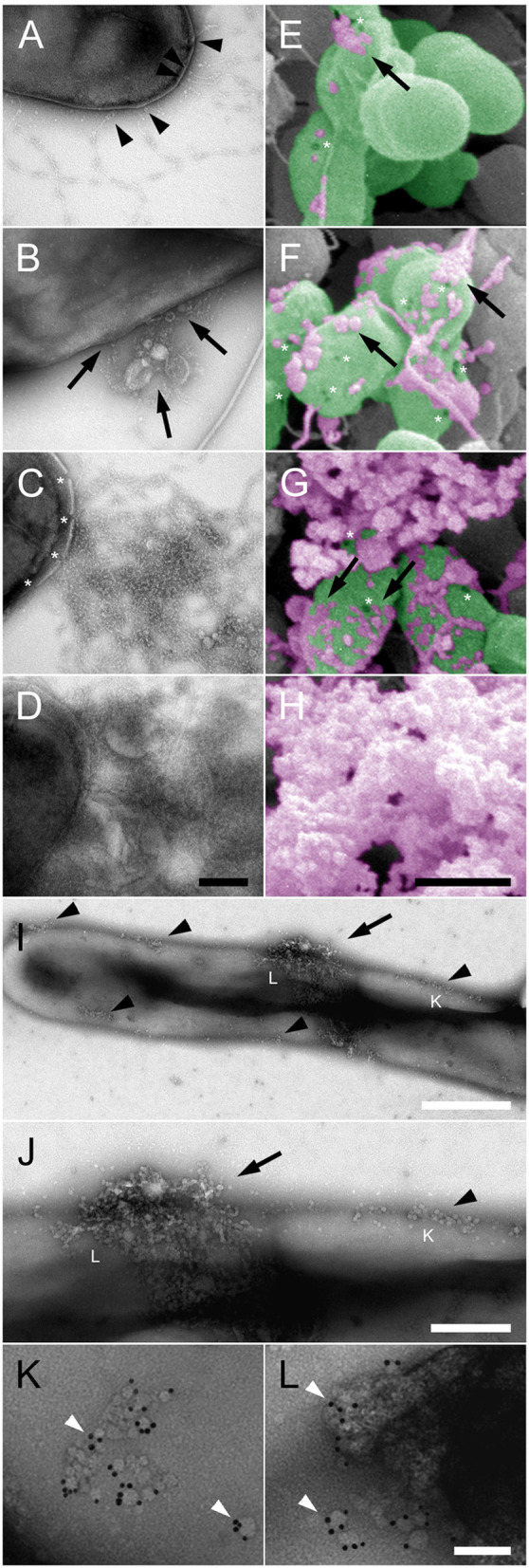
Pore formation and membrane blebbing as early events in collagen VI-induced NTHi membrane rupture. NTHi bacteria were incubated with collagen VI for 0 min **(A,E)**, 30 min **(B,F)**, 60 min **(C,G)**, and 120 min **(D,H)**, and visualized by transmission **(A–D)** and scanning **(E–H)** electron microscopy. Collagen VI microfibrils **(A–H**, arrowheads) adhere to the bacterial surface and induce membrane blebbing **(A–H)**, arrows. Subsequently, membrane rupture (asterisks) leads to large scale exudation of intracellular contents and destruction of the bacteria. In **(E–H)** bacteria are indicated in green, and cytoplasmic exudates in purple pseudocolours. The scale bars represent 200 nm **(A–D)** and 500 nm **(E–H)**. **(I,J)**, overviews at different magnifications of NTHi incubated with collagen VI for a few minutes. Small plasma membrane blebs rapidly accumulate on the bacterial surface (arrows). **(K,L)**, at higher magnification groups of membrane pores and membrane blebs are visible, colocalized with gold-labeled antibodies against the collagen VI VWA domains. The positions of **(K)** and **(L)** are indicated in **(I,J)** by respective letters. The scale bars represent 500 nm **(I)**, 200 nm **(J)**, and 50 nm **(K,L)**.

At higher magnification and resolution the different electron microscopy techniques unraveled more details of the early events preceding large-scale NTHi membrane permeabilization (Figure 2). Initially, beaded collagen VI microfibrils targeted the bacterial surface by binding of the VWA domains to the plasma membrane (Figure 2A, arrowheads). Subsequently, membrane blebs (arrows) and membrane pores (asterisks) became visible all over the bacterial surface, followed by cytoplasmic exudation (Figures 2B–H). The earliest observable change in overall bacterial membrane appearance were groups of small membrane pores and loose aggregates of small membrane blebs (Figures 2I,J, black arrowheads, Figure 2K), which then appeared to coalesce into larger aggregates (Figure 2L). The collagen VI VWA domains were frequently observed in the vicinity of such groups of membrane pores and blebs and visualized by gold-labeled anti-VWA antibodies (Figures 2K,L).

### Targeting of the NTHi surface adhesins PE and hap by collagen VI microfibrils *in vitro*

**Figure 3:**
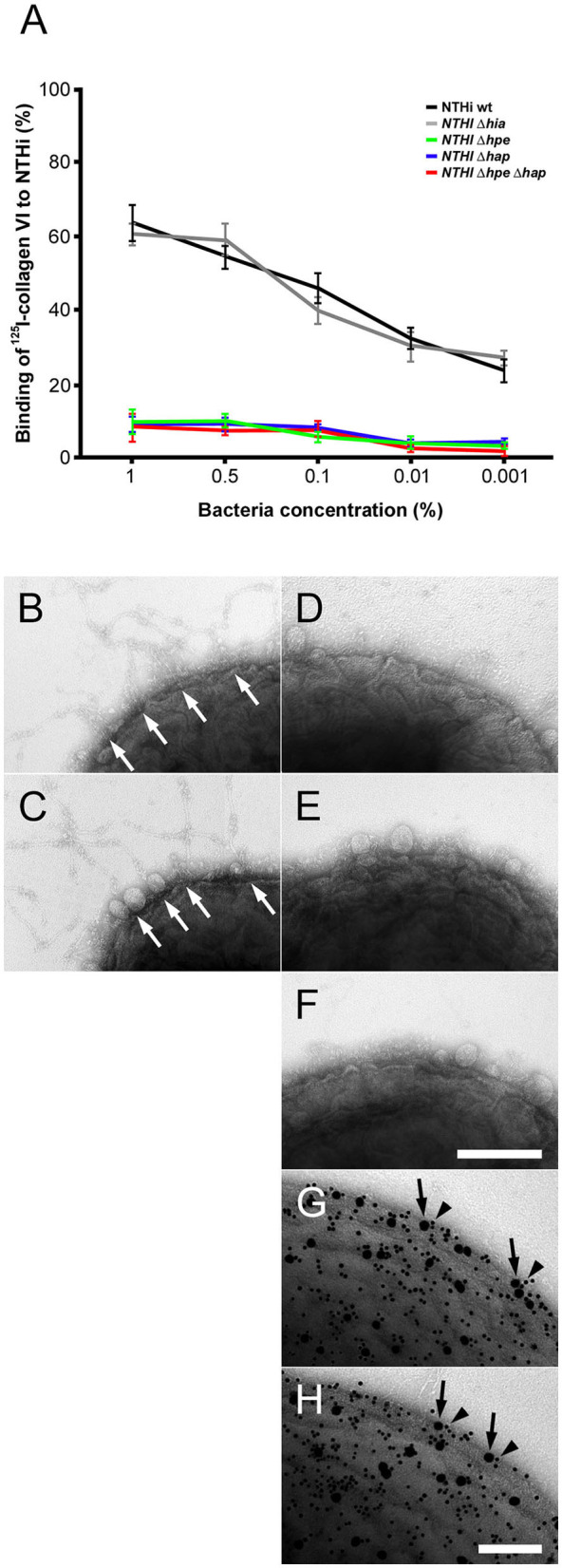
Targeting of NTHi surface adhesins PE and Hap by collagen VI VWA domains. **(A)** Titration of bacterial solutions with radiolabeled collagen VI microfibrils. Serial dilutions of bacteria were used: 1% (2 × 10^9^ cfu/ml), 0.5% (1 × 10^9^ cfu/ml), 0.1% (2 × 10^8^ cfu/ml), 0.01% (2 × 10^7^ cfu/ml), and 0.001% (2 × 10^6^ cfu/ml). Wild type bacteria are compared to isogenic mutants as indicated. **(B–F)** negative staining and transmission electron microscopy of collagen VI networks bound to the bacterial surface. Wild type **(B)** and Δ*hia* **(C)** bacteria interact with collagen VI (arrows) as opposed to Δ*hpe* **(D)**, Δ*hap* **(E)**, and Δ*hpe*Δ*hap* **(F)**. PE **(G)** and Hap **(H)** are frequently colocalized with collagen VI on the bacterial surface as visualized by antibodies conjugated with 5 nm (PE and Hap, arrowheads) and 10 nm (collagen VI, arrows) colloidal gold, respectively. The scale bars represent 200 nm **(B–F)** and 100 nm **(G,H)**.

In order to identify NTHi surface adhesins for primary adhesion to collagen VI we performed radioligand binding studies (Figure 3). Intact full-length collagen VI microfibrils were purified from bovine cornea and radiolabeled with ^125^I. Diluted samples were added to NTHi suspensions in physiological saline and the relative amount of radioactivity associated with the bacterial pellet was determined (Figure 3A). Collagen VI strongly bound to NTHi wild type (wt) and a control isogenic mutant lacking Hia (NTHi Δ*hia*). In contrast, comparison of NTHi mutants devoid of PE (Δ*hpe*), Hap (Δ*hap*), and the double mutant Δ*hpe*/Δ*hap* revealed that the absence of these two adhesins abolished the ability to recruit collagen VI. Similar observations were made by negative staining and transmission electron microscopy. Large amounts of beaded collagen VI microfibrils accumulated on the surface of NTHi wt (Figure 3B, arrows) and Δ*hia* (Figure 3C, arrows). In contrast, Δ*hpe* (Figure 3D), Δ*hap* (Figure 3E), and Δ*hpe*Δ*hap* (Figure 3F) mutant bacteria did not exhibit any affinity for microfibrils. In another parallel experimental set up, negative staining and immunoelectron microscopy visualized that PE (Figure 3G, 5 nm gold, arrowheads) and Hap (Figure 3H, 5 nm gold, arrowheads) were frequently colocalized with the collagen VI VWA domains (10 nm gold, arrows) on the bacterial surface. In addition, experiments with *E. coli* laboratory strains with plasmids expressing Hap, PE or Hia were also performed (Supplementary Figure S1). Taken together, NTHi adhesins PE and Hia significantly promoted bacterial binding to collagen VI, whereas Hia was not involved in these interactions.

### Targeting of the NTHi surface adhesins PE and hap by the collagen VI VWA domains *in vitro*

**Figure 4:**
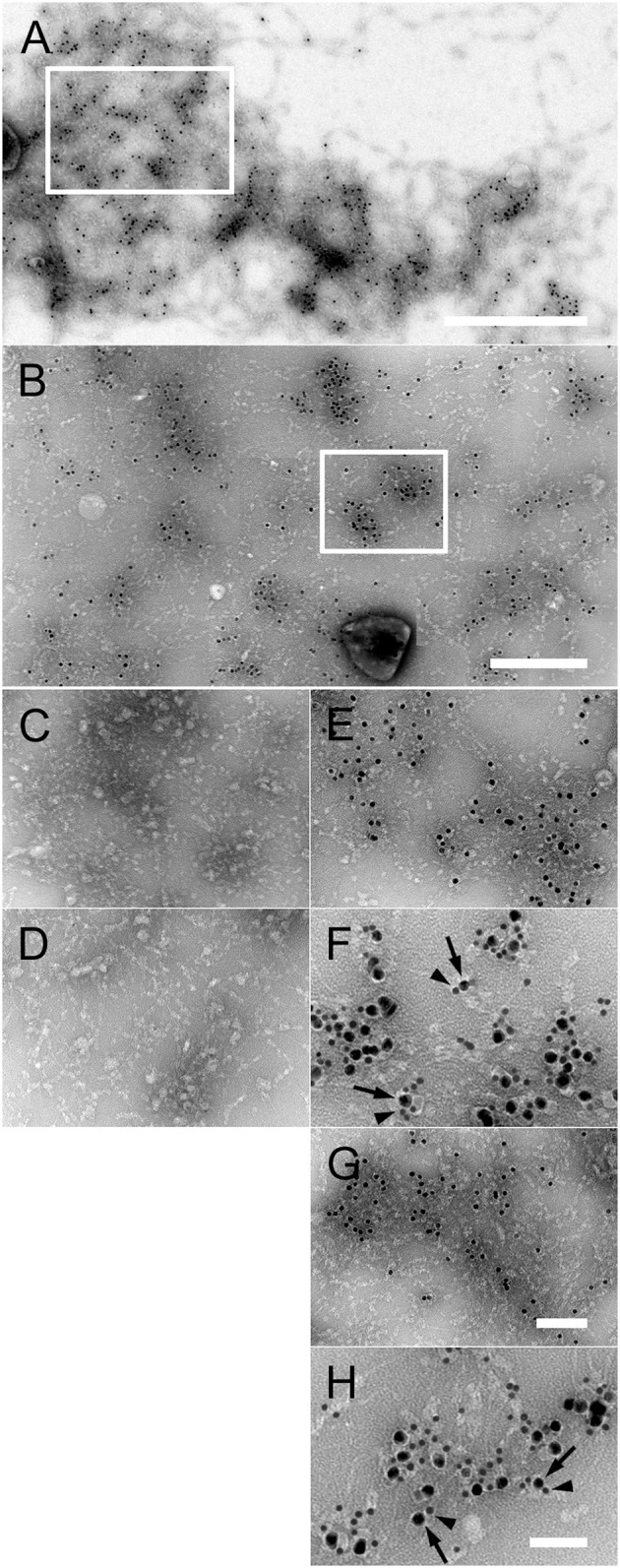
Interaction of purified PE and Hap with collagen VI networks *in vitro*. **(A)** Overview of extended native collagen VI networks from bovine cornea after incubation with gold-labeled PE. The 10 nm gold-PE conjugates are barely visible as black dots. The scale bar represents 500 nm. **(B)** Enlargement of an area corresponding to the frame in **(A)** exhibiting extensive binding of 10 nm gold-labeled PE to the collagen VI network. Scale bar, 250 nm. **(C–H)** Enlargements of collagen VI networks incubated with the different gold-labeled NTHi surface proteins as indicated by the frame in **(B)**. **(C)** Collagen VI alone, **(D)** Hia, **(E)** PE, **(F)** colocalisation of PE (10 nm) with collagen VI VWA domains (5 nm), **(G)** Hap, **(H)** colocalisation of Hap (10 nm) with collagen VI VWA domains (5 nm). The scale bars represent 100 nm **(C–E,G)** and 50 nm **(F,H)**.

Based on our results on NTHi adhesins and collagen VI (Figure 3), we were tempted to gain a more detailed insight into the molecular interactions between PE, Hap, and collagen VI in a system with purified components. Collagen VI microfibrillar networks were incubated at high concentrations, resembling an *in vivo* situation, with different purified NTHi adhesins conjugated to colloidal gold (Figure 4, Supplementary Figure S2). Extended multimolecular complexes were formed between PE and collagen VI networks (Figures 4A,B,E,F). Similar observations were made for Hap (Figures 4G,H). In contrast, Hia was rarely observed as a ligand for collagen VI networks (Figure 4D) and electron micrographs thereof appeared similar to collagen VI microfibrils without added ligand-gold conjugates (Figure 4C). Immunoelectron microscopy with gold-labeled collagen VI antibodies showed that PE (Figure 4F, arrowheads) and Hap (Figure 4H, arrowheads) were frequently colocalized with the collagen VI VWA domains (arrows). At lower concentrations, gold-labeled PE (Supplementary Figure S2A, arrows) and Hap (Supplementary Figure S2B, arrows) were visualized in close contact with the globular collagen VI VWA domains (arrows). In contrast, Hia sparsely bound to collagen VI (Supplementary Figure S2C, arrow). Instead, the bulk of Hia molecules appeared randomly distributed in the background (arrowheads). In another experimental set-up further evidence for the molecular details of the PE and Hap interactions with collagen VI was obtained. Preincubation with anti-VWA antibodies abolished the interactions between PE, Hap, and collagen VI VWA (Supplementary Figures S2D–F). Gold conjugates of PE (Supplementary Figure S2D), Hap (Supplementary Figure S2E), and Hia (Supplementary Figure S2F) were almost exclusively found unbound outside the collagen VI microfibrils (arrowheads) and only minute amounts of molecules were observed in complex with the collagen VI VWA domains (arrow). Similar observations were made for non-labeled PE, Hap, and Hia (Supplementary Figures S2G–I).

### NTHi adherence to the airway mucosa and human pulmonary fibroblasts *ex vivo*

**Figure 5:**
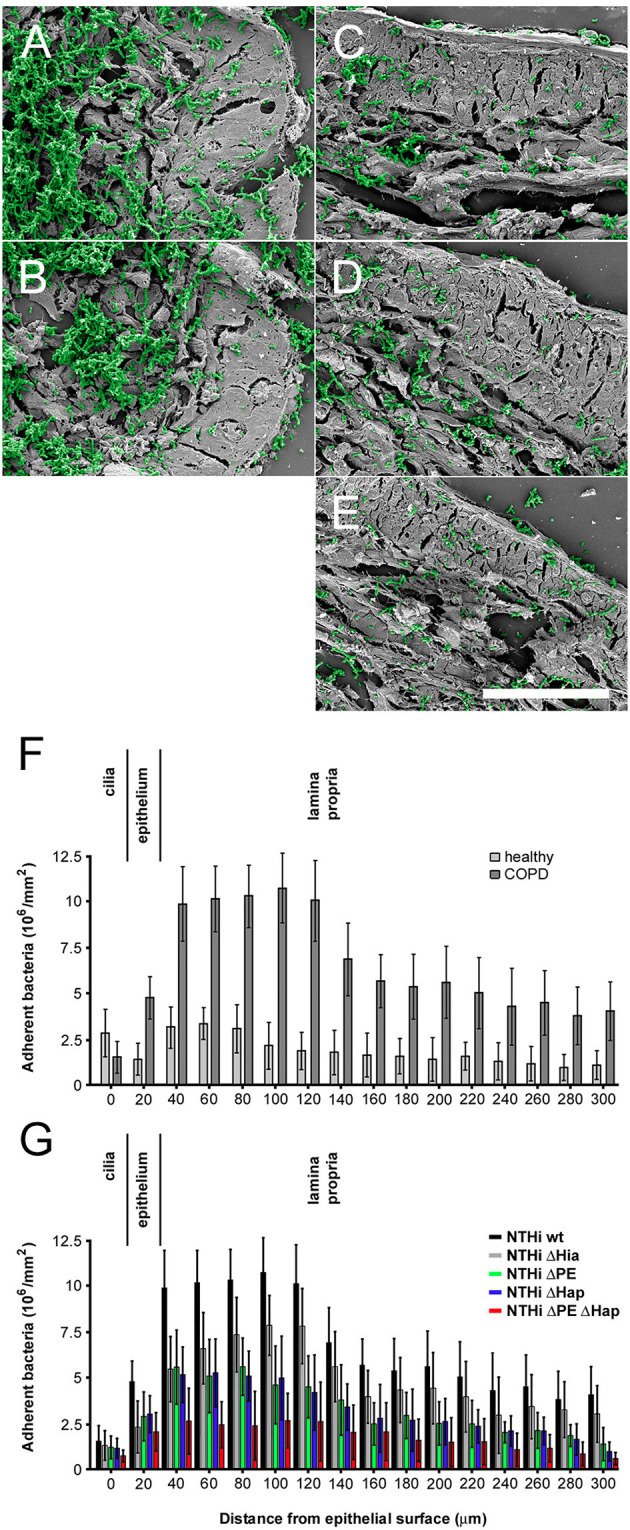
Colonization of NTHi in COPD airways *ex vivo*. Scanning electron micrographs of paraffin sections of airway biopsies from COPD patients inoculated with NTHi wild type **(A)**, Δ*hia* **(B)**, Δ*hpe* **(C)**, Δ*hap* **(D)**, and Δ*hpe*Δ*hap* **(E)**. In COPD, considerable numbers of wild type and Δ*hia* bacteria are observed bound to the airway submucosa **(A,B)** as compared to bacteria lacking PE **(C)**, Hap **(D)**, or both adhesins **(E)**. Bacteria are highlighted in green pseudocolour. The scale bar represents 50 μm. **(F,G)** Quantitative evaluation of adherent bacteria as indicated.

Airway biopsies from COPD patients undergoing surgery for suspected lung cancer were challenged with NTHi wt and isogenic mutant bacteria, washed extensively and prepared for high resolution scanning electron microscopy (Figure 5). Tissue areas were selected as far from the solid tumor as possible to correspondingly decrease the risk of inflammatory influence despite it could not be fully excluded. Electron micrographs exhibited large fields of bronchopulmonary tissue preparations with massive infection of the lamina propria with NTHi wt (Figure 5A) and Δ*hia* mutants (Figure 5B). These observations are in accordance with the previously described upregulation of collagen VI targets in the fibrotic extracellular matrix (24). In contrast, isogenic mutants lacking PE (Figure 5C), Hap (Figure 5D), or both surface proteins (Figure 5E) resulted in considerably less bacterial load. In airway biopsies from healthy individuals an overall similar pattern of bacterial adherence was observed, but at a considerably lower level (results not shown). Thus, we performed a quantitative evaluation of 50 cellular profiles of NTHi adhesion to healthy and COPD specimens (Figures 5F,G). In airways from healthy individuals, bacteria bound to the ciliated epithelial lining to some extent. There were moderate amounts of bacteria binding to intracellular targets and some more extensive adherence to the subepithelial lamina propria (Figure 5G, healthy). In contrast, in biopsies from COPD patients, overall NTHi adherence was greatly enhanced (Figure 5G, COPD). The most efficient infection was observed for NTHi wt and Δ*hia* mutant pathogens, whereas Δ*hpe*, Δ*hap*, and the double mutant Δ*hpe*Δ*hap* bound to a considerably lesser extent (Figure 5G). The most pronounced binding to the extracellular matrix was observed in the collagen VI-rich region distant to the basement membrane, which is in accordance with recent findings (24). Similar observations were made in pulmonary fibroblast cell culture after inoculation with NTHi (Supplementary Figure S3). Colonies of bacteria frequently adhered to extracellular matrix fibrils secreted by COPD fibroblasts (Supplementary Figure S3F) and to a lesser extent to healthy fibroblasts (Supplementary Figure S3A). In analogy to binding behavior on airway biopsies, bacteria lacking PE (Supplementary Figures S3B,G), Hap (Supplementary Figures S3C,H), or both PE and Hap (Supplementary Figures S3E,J) adhered considerably less. Similar observations were made upon preincubation of the system with antibodies against collagen VI (Supplementary Figures S3K,P), PE (Supplementary Figures S3L,G), Hap (Supplementary Figures S3M,R), or against both adhesins (Supplementary Figures S3O,T) that significantly reduced bacterial adherence. Taken together, these results reveal the physiological importance of the molecular interplay of the PE and Hap with collagen VI for optimized pathogen-dependent adherence in invasive bronchial airway infection in patients suffering from COPD.

### NTHi adheres to collagen VI microfibrils in the airway mucosa *in vivo*, followed by membrane disruption and killing

**Figure 6:**
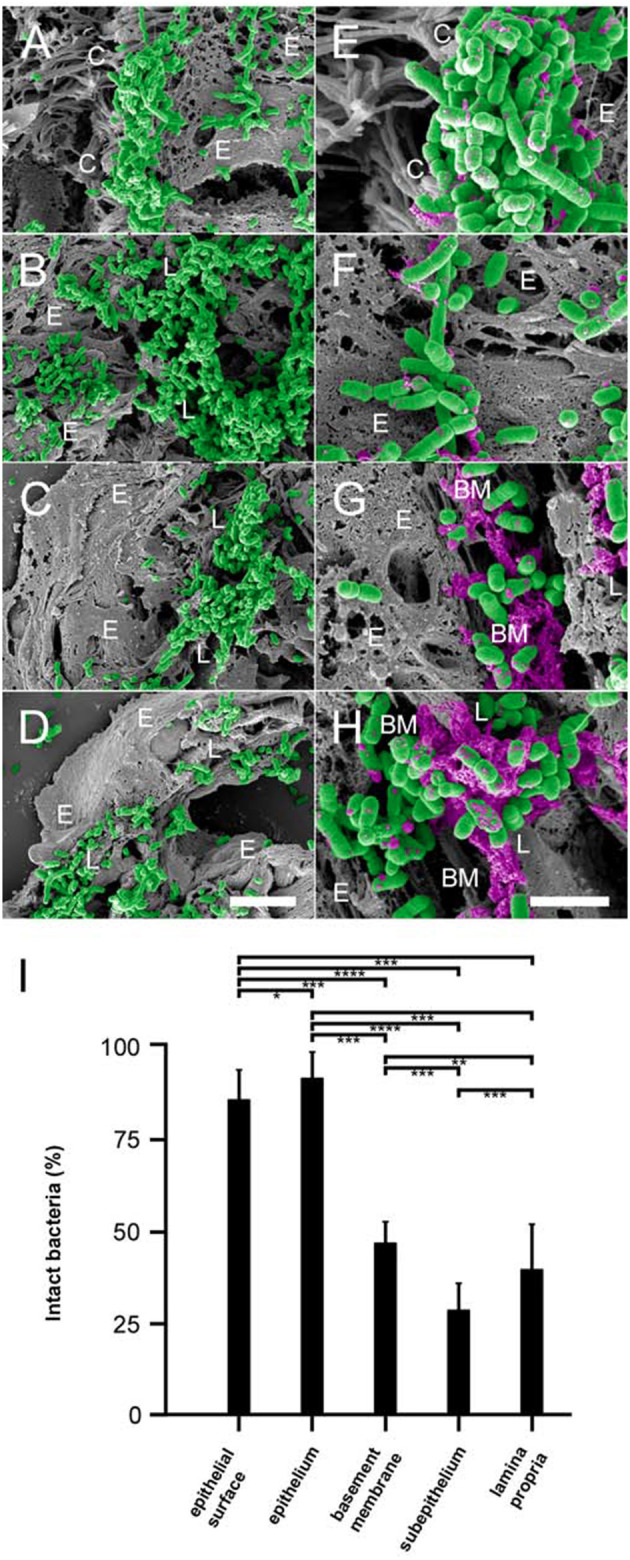
Colonization of pulmonary compartments by NTHi and antimicrobial activity of the lamina propria *in vivo*. Paraffin sections of murine COPD lungs inoculated with NTHi by intratracheal challenge. Scanning electron microscopy of large airways reveals extensive binding of bacteria to the epithelial surface **(A)** and the lamina propria **(B)**. Similar observations were made for small airways **(C)** and alveoli **(D)**, except that bacteria colonize the subepithelial lamina propria and bind only sparsely to the epithelial surface. **(E–H)** Higher magnification of the same areas reveals killing of NTHi bacteria upon exposure to the epithelial surface **(E)**, the epithelial cell layer **(F)**, the subepithelial basement membrane **(G)**, and the lamina propria **(H)**. Bacteria are highlighted in green and cytoplasmic exudates in purple pseudocolour. Cellular and tissue structures are indicated as follows; C, epithelial cilia; E, epithelial cell; L, lamina propria; BM, basement membrane. The scale bars represent 5 μm **(A–D)** and 2 μm **(E–H)**. **(I)** Quantitative evaluation of bacterial killing as indicated.

In order to investigate the *in vivo* relevance of our findings on NTHi and collagen VI, we allowed NTHi wt and isogenic mutants to adhere to the bronchopulmonary system of COPD mice. Animals were inoculated with pathogens by intratracheal challenge, followed by incubation for 30 min. The lungs were excised and embedded in paraffin. Sections were taken from different locations in the tissue in order to retrieve suitable areas of infection. Specimens were examined by high resolution scanning electron microscopy (Figure 6). Large amounts of NTHi adhered to the lamina propria of the large airways (Figure 6B), and to some extent to the ciliated epithelial surface (Figure 6A). Similarly, extensive bacterial adherence was observed to the lamina propria of small airways (Figure 6C) and alveoli (Figure 6D). As opposed to the large airways, only minute amounts of bacteria adhered to the epithelial surface of small airways and alveoli. Interestingly, already after 30 min exposure to the bronchopulmonary mucosa, bacteria were killed to variable extent. Both at the epithelial surface (Figure 6E) and in the epithelial cell layer (Figure 6F) moderate amounts of killed bacteria with visible cytoplasmic exudates were present. In contrast, the collagen VI-containing subepithelial extracellular matrix exhibited a considerably higher killing potential (Figures 6G,H). In the basement membrane area (Figure 6G), large amounts of killed bacteria were observed. The largest relative amounts of killed pathogens were present in the subepithelial space distant to the basement membrane and in the deeper layers of the lamina propria (Figure 6H). These observations were confirmed by a quantitative evaluation of bacterial killing in the different pulmonary compartments (Figure 6I). Transmission immunoelectron microscopy of inoculated murine airways frequently visualized NTHi bacteria in contact with extracellular collagen VI fibrils in murine airway samples (Supplementary Figures S4A–C). Similar observations were made in COPD patient samples obtained by bronchoscopy (Supplementary Figures S4D–F). The fraction of NTHi particles with disrupted structural appearance was considerably higher in the vicinity of collagen VI microfibrils than in other locations of the extracellular space, e.g., basement membrane structures, collagen I fibrils, or elastic fibers (Supplementary Figure S5). Binding to collagen VI *in vivo* was mediated by the surface adhesins PE (Supplementary Figures S4B,E) and Hap (Supplementary Figures S4C,F). In contrast, Hia was not colocalized with collagen VI fibrils (data not shown). In airway samples from infected healthy control mice an overall similar pattern of bacterial adherence and killing was observed, but at considerably lower levels (results not shown).

### Time dependent killing of NTHi *in vivo* and protective properties of the surface adhesin PE

**Figure 7:**
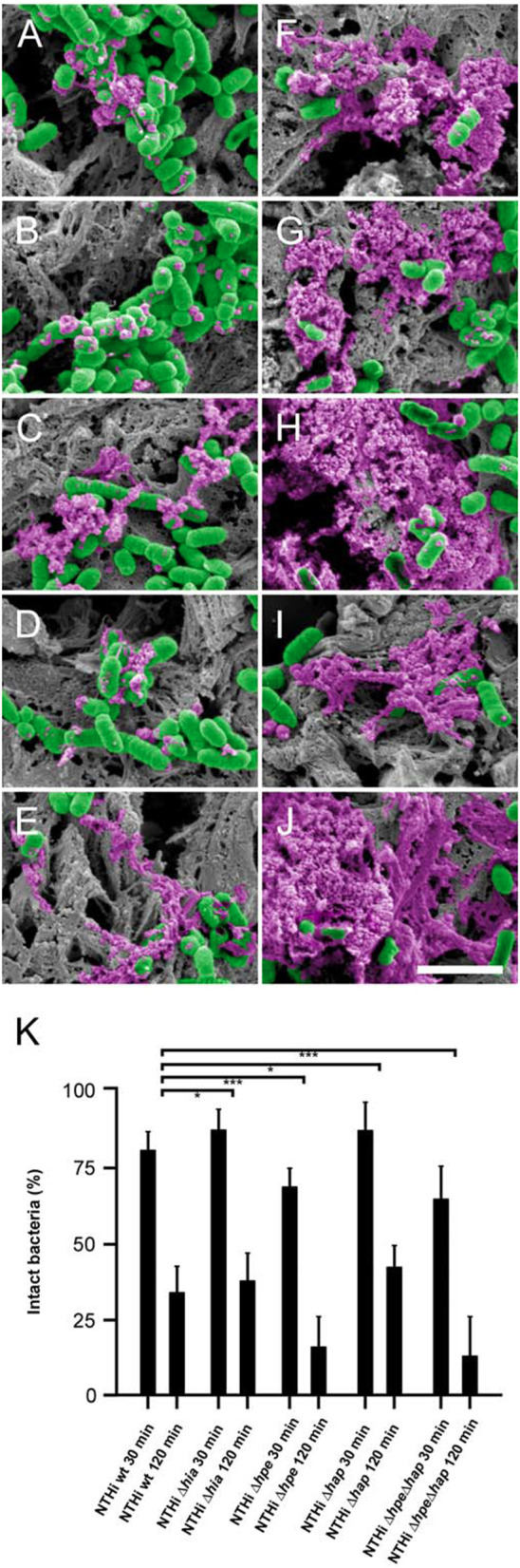
Time-dependent NTHi killing *in vivo* and partial protection by PE. COPD mice were challenged by intratracheal inhalation with NTHi wild type **(A,F)**, Δ*hia* **(B,G)**, Δ*hpe* **(C,H)**, Δ*hap* **(D,I)**, or Δ*hpe*Δ*hap* **(E,J)**. Bacteria present in the airways after 30 min **(A–E)** and after 2 h **(F–J)** were analyzed by scanning electron microscopy. Bacteria are highlighted in green and cytoplasmic exudates in purple pseudocolour. The scale bar represents 2 μm. **(K)** Quantitative evaluation of bacterial killing as indicated.

COPD mice were inoculated with NTHi wt and Δ*hia*, Δ*hpe*, Δ*hap*, and Δ*hpe*/Δ*hap* mutants by intratracheal challenge. After incubation for 30 min and 2 h, respectively, lungs were excised and embedded in paraffin. Specimens were examined by high resolution scanning electron microscopy (Figure 7). The pathogens adhered specifically to the extracellular matrix of the airway mucosa and were killed in a time-dependent manner. After 30 min 15–20% of the pathogens expressing the surface adhesin PE, i.e., NTHi wt (Figure 7A), Δ*hia* (Figure 7B), and Δ*hap* (Figure 7D) exhibited signs of cytoplasmic exudation. In contrast, the absence of PE on the bacterial surface lead to more pronounced membrane rupture and exudation. Approximately 25–30% of the Δ*hpe* (Figure 7C) and Δ*hpe*Δ*hap* (Figure 7E) mutant bacteria were affected by enhanced killing. Correspondingly, after 2 h exposure to the killing potential of the lamina propria app. 80% of the pathogens lacking PE were killed (Δ*hpe*, Figure 7H; Δ*hpe*Δ*hap*, Figure 7J) as compared to 60–65% of bacteria expressing this adhesin (NTHi wt, Figure 7F), Δ*hia* (Figure 7G), and Δ*hap* (Figure 7I). These findings were confirmed by quantitative evaluation of bacterial killing as indicated in Figure 7K. Similar observations were made for NTHi wt and mutant bacteria adhering to human pulmonary fibroblasts from COPD patients (Supplementary Figure S6) and murine COPD lungs (Supplementary Figure S7).

## Discussion

In the present work we show that collagen VI displays adhesive and bactericidal properties against NTHi in the airway mucosa *in vivo*. To our knowledge, this collagen is the first described extracellular matrix holoprotein with such bioactive effects, conferring innate immune protection to the bronchopulmonary system. One likely mechanism of action is membrane-disrupting activity, causing lysis and rapid clearance of the pathogens. Our major findings were (i) the identification of a novel role of two NTHi surface adhesins, PE and Hap, as collagen VI adhesins *in vivo*, (ii) collagen VI promotes bacterial killing *in vivo* in the lamina propria of large and small airways and alveoli, (iii) the bactericidal action occurs by membrane rupture and cytoplasmic exudation, and (iv) the finding that PE confers partial protection against this killing effect.

Despite worldwide efforts, Gram-negative bacterial infections remain a leading global health issue. They are a common cause of sepsis and are increasingly contributing to the development of resistance against conventional antibiotics. Non-typeable *Haemophilus influenzae* (NTHi) is an important pathogen in mucosal infections and exacerbations of COPD (chronic obstructive pulmonary disease) (8). Since the introduction of the conjugated *H. influenzae* b (Hib) vaccine, the incidence of Hib invasive infection has decreased dramatically. Instead, NTHi has become more prevalent and constitutes most invasive *H. influenzae* infection (2, 42–44). It readily affects individuals with co-morbidities or the elderly who are otherwise in good health, and is often clinically severe. Immunocompromised individuals with underlying co-morbidities are also susceptible and invasive infection is associated with high morbidity and fatality rates. Thus, identifying and understanding regulatory processes that fuel the carefully orchestrated interplay of NTHi pathogenesis and host defense responses is inherently challenging.

The human bronchopulmonary system is constantly being exposed to a myriad of potentially harmful microbes. The ability to adhere and adapt to the respiratory mucosa plays a key role for the pathogenesis of NTHi to establish persistent infection and colonization. As a counteracting measure, the host has evolved appropriate immune response strategies to facilitate pathogen clearance and thus minimize the risk for persistent infection (45, 46).

The epithelial cell barrier serves as a first line of host defense by detecting pathogen-associated molecular patterns (PAMPs) and activating downstream biological pathways required for shaping the innate immune/inflammatory response. Underneath the epithelial cell lining the pathogen invaders face yet another line of defense, conferred by extracellular matrix molecules with innate immune properties. However, the spatial and temporal events associated with the highly dynamic host-pathogen crosstalk during infection and inflammation have not yet been fully characterized. Nor is it clearly understood why host immune responses often fail to clear the bacteria from the lower respiratory tract. Therefore, we were tempted to monitor the early molecular events during the first 2 h of infection between invading NTHi and their substrate, collagen VI, in the airway mucosa. The initial stage of colonization is characterized by the binding of NTHi to ciliated cells and subsequently to the underlying lamina propria in areas of epithelial denudation. Spatial and temporal profiling of bacterial infection and survival by high resolution scanning electron microscopy reveals significant targeting of collagen VI in the airway extracellular matrix by NTHi bacteria. Concurrently, the adhesive collagen VI substrates efficiently contain and rapidly kill the pathogen invaders like molecular fly paper. Notably, as part of our screening for profiles of infection and bactericidal activity, we observed that these parameters followed the collagen VI expression patterns in the lamina propria.

Collagen VI is an intriguing multifunctional extracellular matrix molecule with diverse bioactive properties. It contains uncharacteristically large N- and C-terminal non-collagenous regions and has the lowest triple helix content among all collagens. It is thus predominantly composed of globular VWA domains which display adhesive and antimicrobial properties at physiological conditions *in vitro* (23, 24). As a consequence of its unique expression and assembly properties and variety of domains, collagen VI forms extended three-dimensional beaded microfibrillar networks in almost all connective tissues. The beads are arranged in 105 nm repeats throughout the microfibrils. Each bead is composed of all the VWA domains of 4 monomers and thus the total number of beads in a given three-dimensional network literally acts as a mine field for pathogen invaders. It is very likely that other multidomain extracellular matrix components display similar innate immune properties (23). Laminin, a major constituent of basement membranes, has recently been reported to contain characteristic peptide stretches with immunomodulating properties (24, 47). Similarly, laminin, fibronectin, and collagens type I and III have been identified as targets for the *Haemophilus ducreyi* ftpA and losB surface adhesins (20). It can be predicted that during the coming years the host connective tissue compartment will emerge as a new branch of innate immunity.

Taken together, in this study we have elucidated functions of collagen VI as an adhesive matrix and bactericidal barrier *in vivo* for NTHi in murine and human airways. In particular, the susceptibility of the extracellular matrix of the airway mucosa to NTHi infection, its innate host defense properties, and the host-pathogen cross talk in COPD are assessed. We conclude that these properties might, at least partially, be conferred by the adhesive and killing properties of the VWA domains of collagen VI. We identified the NTHi surface factors PE and Hap as novel collagen VI adhesins, a hitherto unknown feature, giving the bacteria their adhesive properties. Upon adhesion to collagen VI by interaction of PE and/or Hap with the VWA domains in the airway mucosa, NTHi bacteria are rapidly eliminated by membrane rupture. These findings highlight the many facets of collagen VI as a key factor in bacterial host invasion and innate host defense transmitted by the extracellular matrix. Similar observations have been made for different PE, Hap, and Hia-expressing NTHi strains as well as a variety of pulmonary and other Gram-positive and Gram-negative pathogens [(24), Abdillahi et al. 2018, manuscript in press], introducing collagen VI as a broad-spectrum antimicrobial agent. This molecule renders the host extracellular matrix susceptible to pathogen attachment during distinct stages of infection, holds the pathogens in place and thus prevents them from spreading freely in the host. In a following series of events, this collagen enables the host to clear the infection site from invading pathogens by rapid killing through membrane destabilization. These data suggest significant contributions of collagen VI in mediating NTHi adhesion, containment, and clearance in the bronchopulmonary system. However, the exact molecular mechanisms underlying the antimicrobial properties of collagen VI *in vivo* are still elusive and beyond the scope of this work. A potential molecular mechanism might be the release of antimicrobial host defense peptides from the collagen VI polypeptide chains during the infection and inflammation process. It remains an exciting future challenge to investigate how *in vivo* degradation mechanisms may generate bioactive peptides from collagen VI. Such collagen VI peptides may for instance specifically be released by granulocyte proteases during certain phases in inflammation. Considering this, and the substantial medical needs in this area, innate host defense molecules displaying bactericidal effects offer interesting opportunities. In particular, active antimicrobial collagen VI-derived peptides could be tailored as a novel class of antibiotic drug candidates. The data substantiate that collagen VI, and probably other multifunction extracellular matrix components, playing an important role in innate host defense against NTHi infections, could serve as templates for development of novel antimicrobial treatments.

## Author contributions

SA, KR, and MM contributed to the design and implementation of the research and to the writing of the manuscript. SA, SN, RT, MB, OH, BS, and MM performed the experiments. SA, SN, RT, MB, OH, BS, KR, and MM contributed to the interpretation of the results. LB, JE, and GW-T contributed to the final version of the manuscript.

### Conflict of interest statement

MM was employed by Colzyx AB. The remaining authors declare that the research was conducted in the absence of any commercial or financial relationships that could be construed as a potential conflict of interest.

The reviewer AT and handling Editor declared their shared affiliation.

## Supplementary material

Supplementary Figure S1 Targeting of NTHi surface adhesins PE and Hap by collagen VI on transgenic Escherichia coli surfaces. **(A)** Titration of bacterial solutions with radiolabeled collagen VI microfibrils. Serial dilutions of bacteria were used: 1% (2 × 10^9^ cfu/ml), 0.5% (1 × 10^9^ cfu/ml), 0.1% (2 × 10^8^ cfu/ml), 0.01% (2 × 10^7^ cfu/ml), and 0.001% (2 × 10^6^ cfu/ml). Wild type E. coli bacteria are compared to strains transformed with plasmids containing the hia, hpe, and hap genes as indicated. **(B–I)** negative staining and transmission immunoelectron microscopy of wild type *E. coli* **(B–E)** and bacteria transformed with plasmids carrying the genes hia **(F)**, hpe **(G)**, hap **(H)**, or both hpe, and hap **(I)**. PE **(G,I)** and Hap **(H,I)** are frequently colocalized with collagen VI on the bacterial surface as visualized by antibodies conjugated with 5 nm (PE, arrowheads), 10 nm (Hap, short arrows) and 20 nm (collagen VI, long arrows) colloidal gold, respectively. In contrast, wild type *E. coli* **(B–E)** and *E. coli* expressing Hia **(F)** exhibit only moderate collagen VI binding (long arrows). The scale bar represents 100 nm.

Supplementary Figure S2 Interaction of PE and Hap with collagen VI VWA domains. Native collagen VI microfibrils from bovine cornea were incubated with gold-labeled PE **(A)**, Hap **(B)**, or Hia **(C)**. PE and Hap interact specifically with the globular VWA domains of collagen VI (arrows), whereas the bulk of Hia remains unbound (arrowheads). Upon preincubation with an antibody directed against the collagen VI VWA domains, no or very little binding of PE **(A)**, Hap **(B)**, or Hia **(F)** is observed (arrowheads). The same observations were made after preincubation with non-labeled PE **(G)**, Hap **(H)**, or Hia **(I)**. The scale bar represents 200 nm.

Supplementary Figure S3 Adherence of NTHi to lung fibroblasts of healthy subjects and COPD patients. Fibroblasts from healthy individuals **(A–E,K–O)** and COPD patients **(F–J,P–T)** were stimulated with ascorbate to produce an extracellular collagen matrix. They were inoculated with NTHi wild type **(A,F)** or the mutants Δhia **(B,G)**, Δhpe **(C,H)**, Δhap **(D,I)**, or ΔhpeΔhap **(E,J)**. In parallel experiments, fibroblasts were inoculated with wild type NTHi and antibodies against collagen VI **(K,P)**, Hia **(L,Q)**, PE **(M,R)**, Hap **(N,S)**, or both PE and Hap **(O,T)**. Bacteria are highlighted with green pseudocolour. The scale bar represents 10 μm. **(U,V)** evaluation of adherent bacteria at the cell surface and in the extracellular matrix of healthy **(U)** and COPD **(V)** fibroblasts as indicated in the figure.

Supplementary Figure S4 PE and Hap on the NTHi surface bind to collagen VI-containing extracellular matrix fibrils *in vivo*. **(A–C)** Ultrathin sections of fibroblasts inoculated with NTHi bacteria adhering to the extracellular collagen matrix. **(A)** overview, **(B)** PE, and **(C)** Hap (both labeled with 10 nm gold particles) which are frequently colocalized with collagen VI (5 nm gold) in the extracellular collagen matrix as visualized by antibody-gold conjugates. **(D–E)** NTHi identified in the airway submucosa in biopsies of COPD patients with severe COPD (GOLD stage IV). **(D)** overview, **(E)** PE and **(F)** Hap (both labeled with 10 nm gold) colocalized with collagen VI (5 nm gold). F, Fibroblast; B, bacteria; C, extracellular collagen matrix. The scale bars represent 1 μm **(A,D)** and 100 nm **(B,C,E,F)**.

Supplementary Figure S5 Collagen VI kills NTHi by membrane permeabilization and rupture *in vivo*. COPD mice were challenged by intratracheal inhalation with NTHi wild type. Bacteria present in the airways after 2 h were analyzed by transmission immunoelectron microscopy. **(A)** intact NTHi bacteria in contact with non-collagenous extracellular matrix structures. **(B)** NTHi with distorted morphology are often observed in contact with collagen VI. Protein E (PE, 10 nm gold, arrows) and collagen VI (5 nm gold, arrowheads) are identified by antibody-gold conjugates. The scale bar represents 500 nm. **(C)** Quantitative evaluation of bacterial killing as indicated.

Supplementary Figure S6 PE protects NTHi against the antimicrobial properties of collagen VI secreted by pulmonary fibroblasts. Fibroblasts from COPD patients were inoculated for 2 h at 37°C with wild type **(A,B)**, Δhia **(C)**, Δhpe **(D)**, Δhap **(E)**, or ΔhpeΔhap NTHi **(F)** alone or in the presence of an antibody against the collagen VI VWA domains **(B)**. In the absence of PE, bacterial killing was more pronounced. Bacteria are highlighted in green and cytoplasmic exudates in purple pseudocolour. The scale bar represents 2 μm. **(G)** quantitative evaluation of bacterial killing.

Supplementary Figure S7 Quantitative evaluation of bacterial killing *in vivo*. Tissue samples of murine control and COPD lungs inoculated with NTHi by intratracheal challenge were examined for the presence of bacteria by viable count assays **(A)**. Similarly, the viability of NTHi bacteria was assessed in fibroblast adhesion cultures **(B)**. **(C)**, time-dependent killing of NTHi bacteria *in vivo* in inoculated mouse lungs as shown by viable count assays of homogenized lung tissue samples.
