# Corrigendum: The pulmonary extracellular matrix is a bactericidal barrier against *Haemophilus influenzae* in chronic obstructive pulmonary disease (COPD): implications for an *in vivo* innate host defense function of collagen VI

**DOI:** 10.3389/fimmu.2023.1283161

**Published:** 2023-09-27

**Authors:** Suado M. Abdillahi, Ramesh Tati, Sara L. Nordin, Maria Baumgarten, Oskar Hallgren, Leif Bjermer, Jonas Erjefält, Gunilla Westergren-Thorsson, Birendra Singh, Kristian Riesbeck, Matthias Mörgelin

**Affiliations:** ^1^ Infection Medicine, Department of Clinical Sciences, Lund University, Lund, Sweden; ^2^ Respiratory Medicine and Allergology, Department of Clinical Sciences, Lund University, Lund, Sweden; ^3^ Airway Inflammation and Immunology, Department of Experimental Medical Science, Lund University, Lund, Sweden; ^4^ Lung Biology, Department of Experimental Medical Science, Lund University, Lund, Sweden; ^5^ Clinical Microbiology, Department of Translational Medicine, Lund University, Malmö, Sweden; ^6^ Colzyx AB, Medicon Village, Lund, Sweden

**Keywords:** antimicrobial activity, bronchopulmonary infection, collagen VI, COPD, extracellular matrix, *Haemophilus influenzae*, innate immunity, pulmonary fibrosis

In the published article, there was an error in **Figure 3** as published. Panel 3D does not show the correct bacterial specimen. The corrected **Figure 3** and its caption “Targeting of NTHi surface adhesins PE and Hap by collagen VI VWA domains. **(A)** Titration of bacterial solutions with radiolabeled collagen VI microfibrils. Serial dilutions of bacteria were used: 1% (2 × 10^9^ cfu/ml), 0.5% (1 × 10^9^ cfu/ml), 0.1% (2 × 10^8^ cfu/ml), 0.01% (2 × 10^7^ cfu/ml), and 0.001% (2 × 10^6^ cfu/ml). Wild type bacteria are compared to isogenic mutants as indicated. **(B–F)** negative staining and transmission electron microscopy of collagen VI networks bound to the bacterial surface. Wild type **(B)** and Δ*hia* **(C)** bacteria interact with collagen VI (arrows) as opposed to Δ*hpe* **(D)**, Δ*hap* **(E)**, and Δ*hpe*Δ*hap* **(F)**. PE **(G)** and Hap **(H)** are frequently colocalized with collagen VI on the bacterial surface as visualized by antibodies conjugated with 5 nm (PE and Hap, arrowheads) and 10 nm (collagen VI, arrows) colloidal gold, respectively. The scale bars represent 200 nm **(B–F)** and 100 nm **(G, H)**.” appear below.

**Figure 3:**
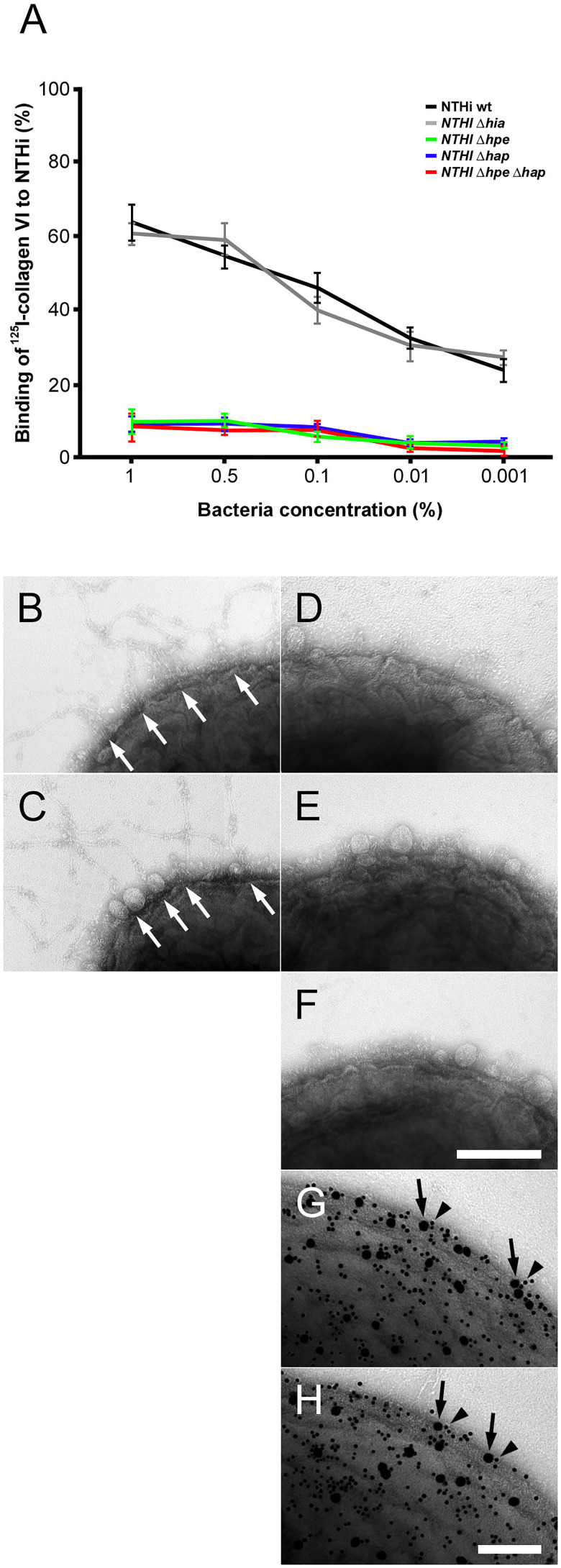
Targeting of NTHi surface adhesins PE and Hap by collagen VI VWA domains. **(A)** Titration of bacterial solutions with radiolabeled collagen VI microfibrils. Serial dilutions of bacteria were used: 1% (2 × 10^9^ cfu/ml), 0.5% (1 × 10^9^ cfu/ml), 0.1% (2 × 10^8^ cfu/ml), 0.01% (2 × 10^7^ cfu/ml), and 0.001% (2 × 10^6^ cfu/ml). Wild type bacteria are compared to isogenic mutants as indicated. **(B–F)** negative staining and transmission electron microscopy of collagen VI networks bound to the bacterial surface. Wild type **(B)** and Δ*hia* **(C)** bacteria interact with collagen VI (arrows) as opposed to Δ*hpe* **(D)**, Δ*hap* **(E)**, and Δ*hpe*Δ*hap* **(F)**. PE **(G)** and Hap **(H)** are frequently colocalized with collagen VI on the bacterial surface as visualized by antibodies conjugated with 5 nm (PE and Hap, arrowheads) and 10 nm (collagen VI, arrows) colloidal gold, respectively. The scale bars represent 200 nm **(B–F)** and 100 nm **(G, H)**.

The authors apologize for this error and state that this does not change the scientific conclusions of the article in any way. The original article has been updated.

