# Supplementary figures and images for: The Pulmonary Extracellular Matrix Is a Bactericidal Barrier Against Haemophilus influenzae in Chronic Obstructive Pulmonary Disease (COPD): Implications for an in vivo Innate Host Defense Function of Collagen VI
Source: Front Immunol. 2018 Aug 31;9:1988. doi: 10.3389/fimmu.2018.01988 (PMC6127292; doi:10.3389/fimmu.2018.01988)

suppl Fig. 1

A

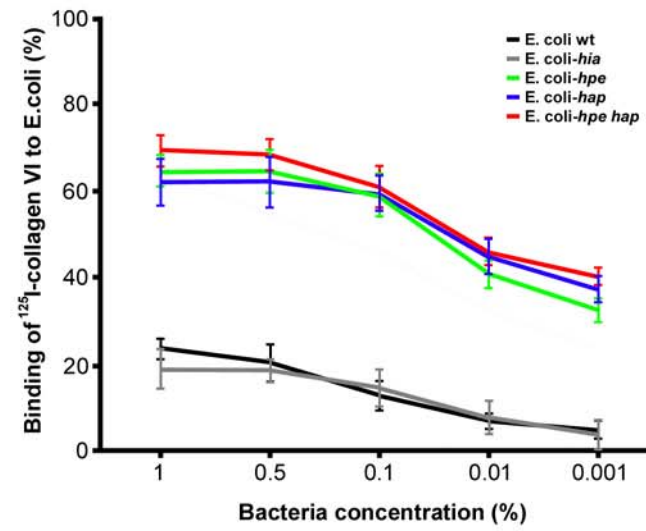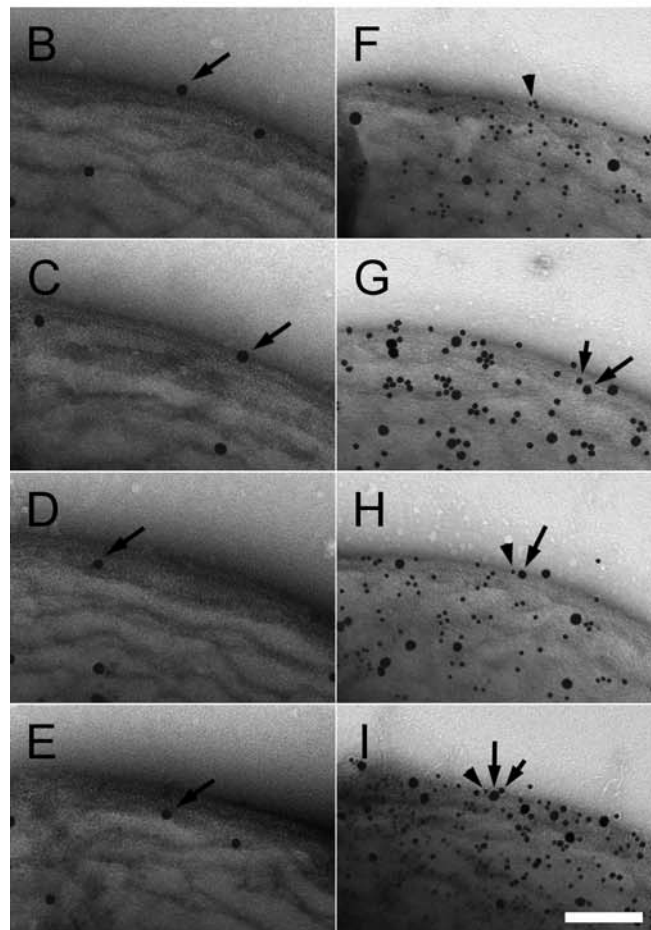

Supplement: Supplementary Figure S1 — Targeting of NTHi surface adhesins PE and Hap by collagen VI on transgenic Escherichia coli surfaces. (A) Titration of bacterial solutions with radiolabeled collagen VI microfibrils. Serial dilutions of bacteria were used: 1% (2 × 10^9^ cfu/ml), 0.5% (1 × 10^9^ cfu/ml), 0.1% (2 × 10^8^ cfu/ml), 0.01% (2 × 10^7^ cfu/ml), and 0.001% (2 × 10^6^ cfu/ml). Wild type E. coli bacteria are compared to strains transformed with plasmids containing the hia, hpe, and hap genes as indicated. (B–I) negative staining and transmission immunoelectron microscopy of wild type E. coli (B–E) and bacteria transformed with plasmids carrying the genes hia (F), hpe (G), hap (H), or both hpe, and hap (I). PE (G,I) and Hap (H,I) are frequently colocalized with collagen VI on the bacterial surface as visualized by antibodies conjugated with 5 nm (PE, arrowheads), 10 nm (Hap, short arrows) and 20 nm (collagen VI, long arrows) colloidal gold, respectively. In contrast, wild type E. coli (B–E) and E. coli expressing Hia (F) exhibit only moderate collagen VI binding (long arrows). The scale bar represents 100 nm. [file Image_1.PDF]

suppl Fig. 2

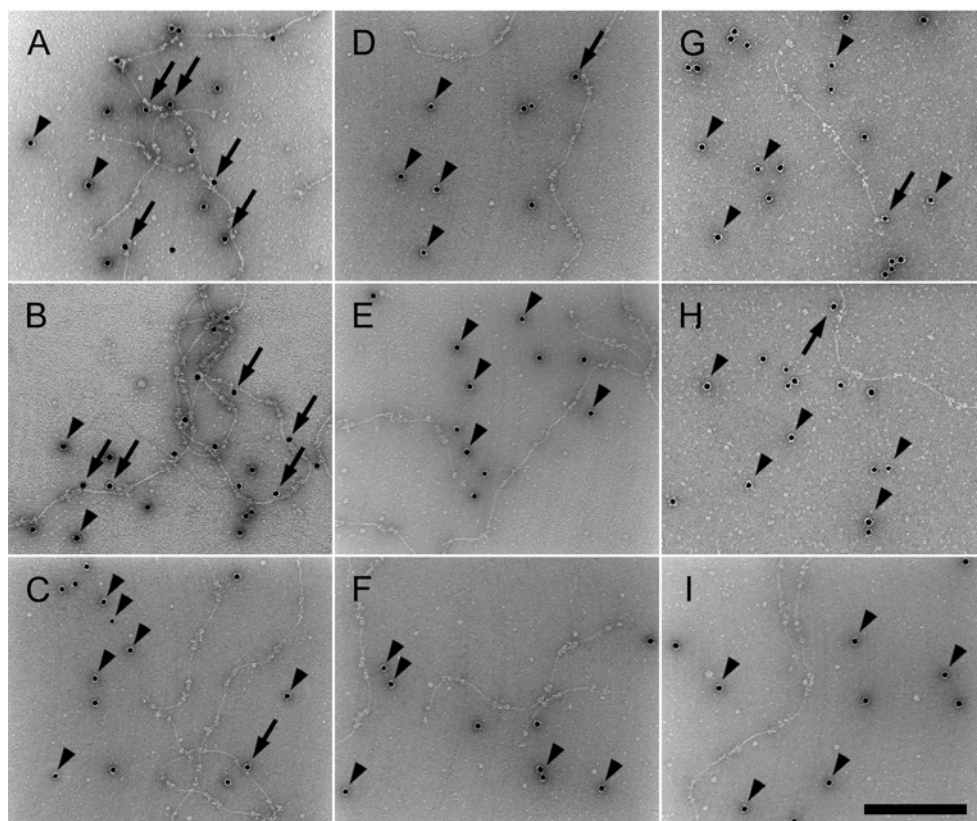

Supplement: Supplementary Figure S2 — Interaction of PE and Hap with collagen VI VWA domains. Native collagen VI microfibrils from bovine cornea were incubated with gold-labeled PE (A), Hap (B), or Hia (C). PE and Hap interact specifically with the globular VWA domains of collagen VI (arrows), whereas the bulk of Hia remains unbound (arrowheads). Upon preincubation with an antibody directed against the collagen VI VWA domains, no or very little binding of PE (A), Hap (B), or Hia (F) is observed (arrowheads). The same observations were made after preincubation with non-labeled PE (G), Hap (H), or Hia (I). The scale bar represents 200 nm. [file Image_2.PDF]

suppl Fig. 3

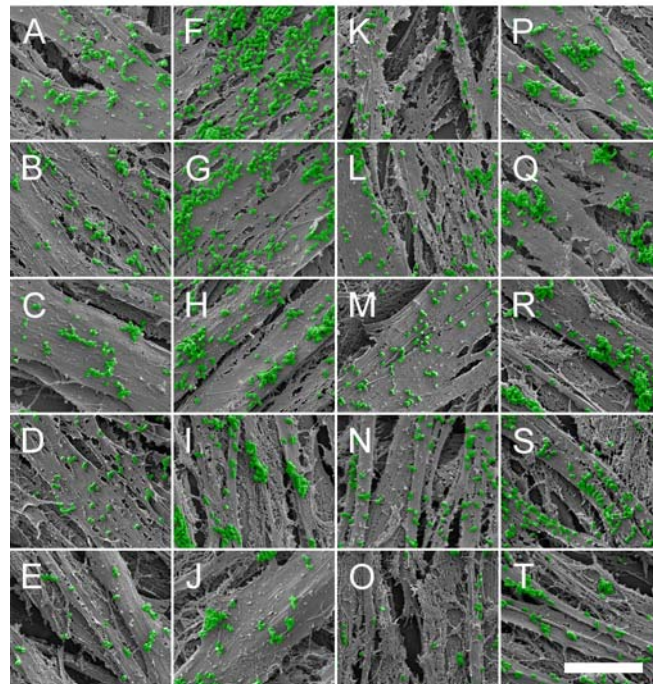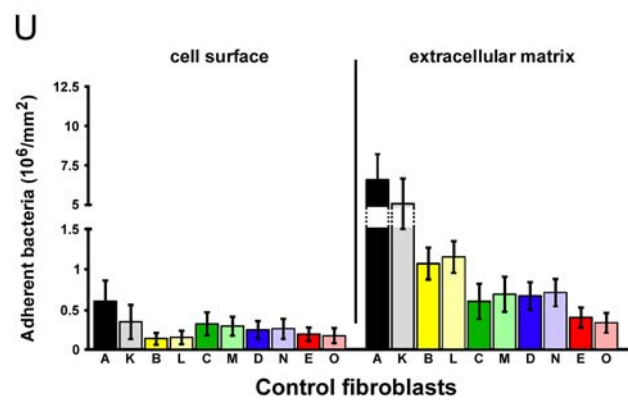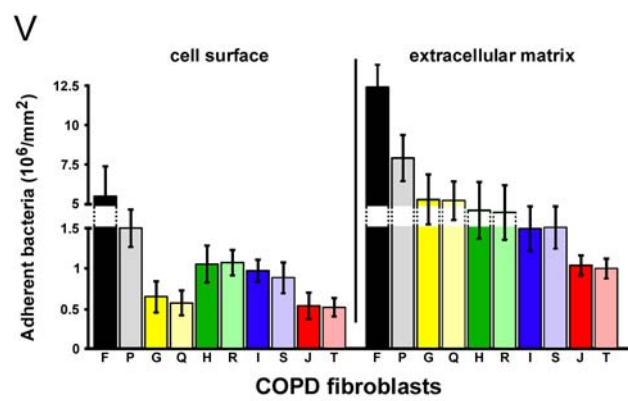

Supplement: Supplementary Figure S3 — Adherence of NTHi to lung fibroblasts of healthy subjects and COPD patients. Fibroblasts from healthy individuals (A–E,K–O) and COPD patients (F–J,P–T) were stimulated with ascorbate to produce an extracellular collagen matrix. They were inoculated with NTHi wild type (A,F) or the mutants Δhia (B,G), Δhpe (C,H), Δhap (D,I), or ΔhpeΔhap (E,J). In parallel experiments, fibroblasts were inoculated with wild type NTHi and antibodies against collagen VI (K,P), Hia (L,Q), PE (M,R), Hap (N,S), or both PE and Hap (O,T). Bacteria are highlighted with green pseudocolour. The scale bar represents 10 μm. (U,V) evaluation of adherent bacteria at the cell surface and in the extracellular matrix of healthy (U) and COPD (V) fibroblasts as indicated in the figure. [file Image_3.PDF]

suppl Fig. 4

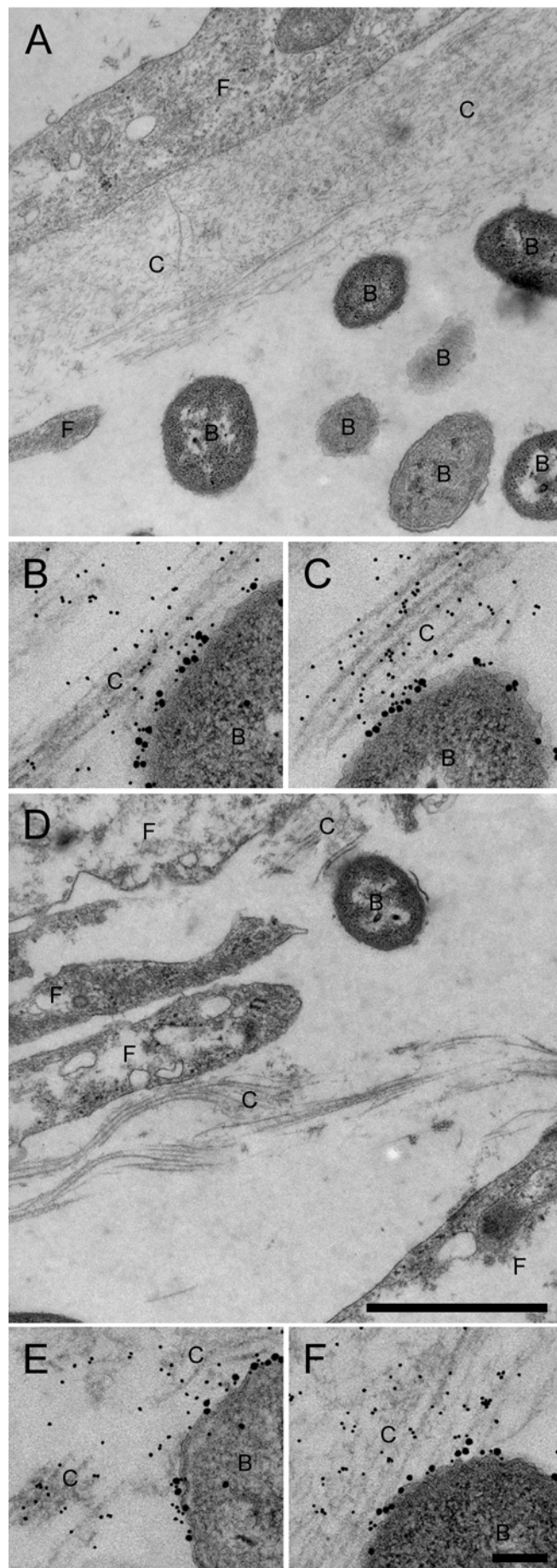

Supplement: Supplementary Figure S4 — PE and Hap on the NTHi surface bind to collagen VI-containing extracellular matrix fibrils in vivo. (A–C) Ultrathin sections of fibroblasts inoculated with NTHi bacteria adhering to the extracellular collagen matrix. (A) overview, (B) PE, and (C) Hap (both labeled with 10 nm gold particles) which are frequently colocalized with collagen VI (5 nm gold) in the extracellular collagen matrix as visualized by antibody-gold conjugates. (D–E) NTHi identified in the airway submucosa in biopsies of COPD patients with severe COPD (GOLD stage IV). (D) overview, (E) PE and (F) Hap (both labeled with 10 nm gold) colocalized with collagen VI (5 nm gold). F, Fibroblast; B, bacteria; C, extracellular collagen matrix. The scale bars represent 1 μm (A,D) and 100 nm (B,C,E,F). [file Image_4.PDF]

suppl Fig. 5

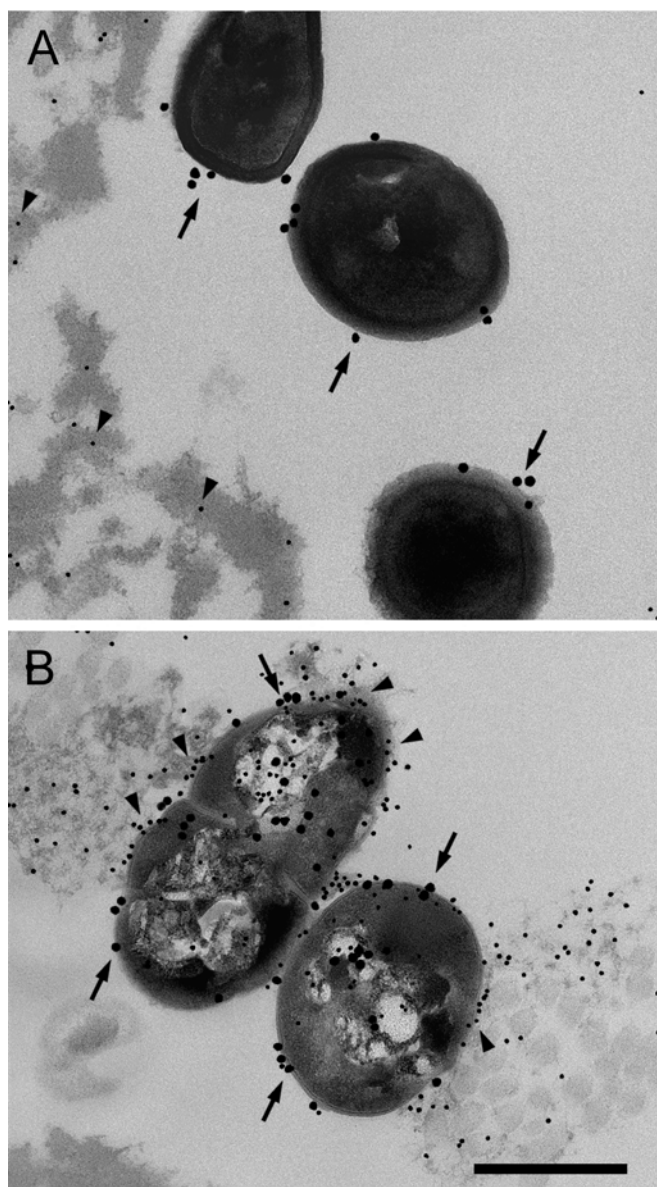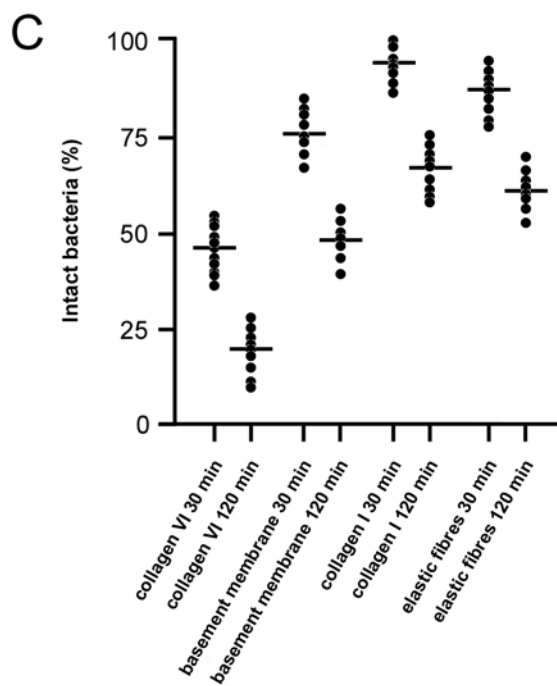

Supplement: Supplementary Figure S5 — Collagen VI kills NTHi by membrane permeabilization and rupture in vivo. COPD mice were challenged by intratracheal inhalation with NTHi wild type. Bacteria present in the airways after 2 h were analyzed by transmission immunoelectron microscopy. (A) intact NTHi bacteria in contact with non-collagenous extracellular matrix structures. (B) NTHi with distorted morphology are often observed in contact with collagen VI. Protein E (PE, 10 nm gold, arrows) and collagen VI (5 nm gold, arrowheads) are identified by antibody-gold conjugates. The scale bar represents 500 nm. (C) Quantitative evaluation of bacterial killing as indicated. [file Image_5.PDF]

suppl Fig. 6

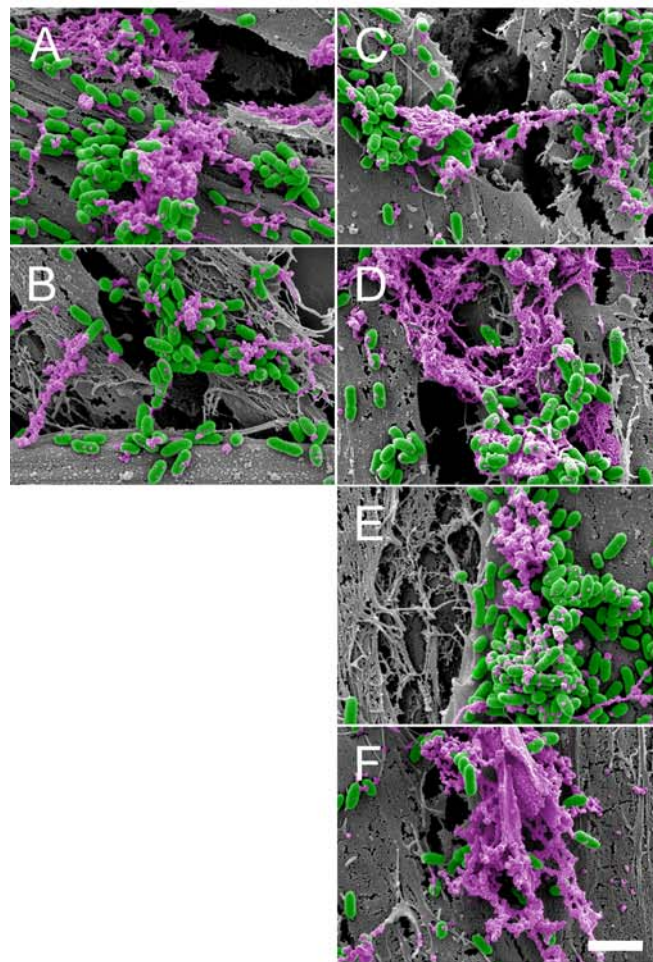

G

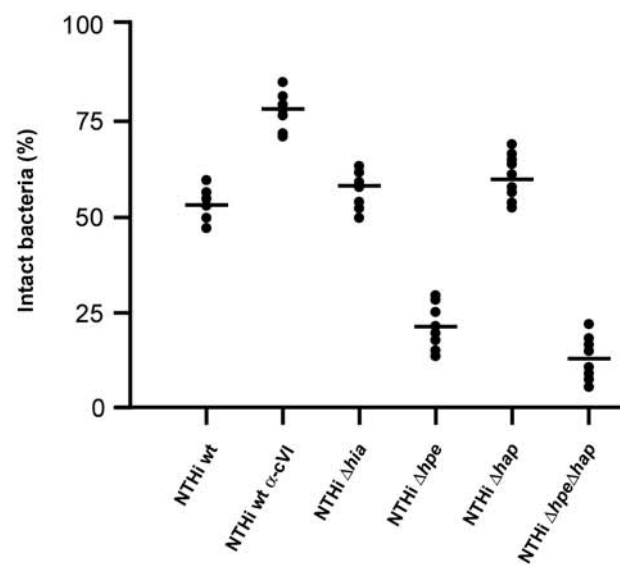

Supplement: Supplementary Figure S6 — PE protects NTHi against the antimicrobial properties of collagen VI secreted by pulmonary fibroblasts. Fibroblasts from COPD patients were inoculated for 2 h at 37°C with wild type (A,B), Δhia (C), Δhpe (D), Δhap (E), or ΔhpeΔhap NTHi (F) alone or in the presence of an antibody against the collagen VI VWA domains (B). In the absence of PE, bacterial killing was more pronounced. Bacteria are highlighted in green and cytoplasmic exudates in purple pseudocolour. The scale bar represents 2 μm. (G) quantitative evaluation of bacterial killing. [file Image_6.PDF]

A

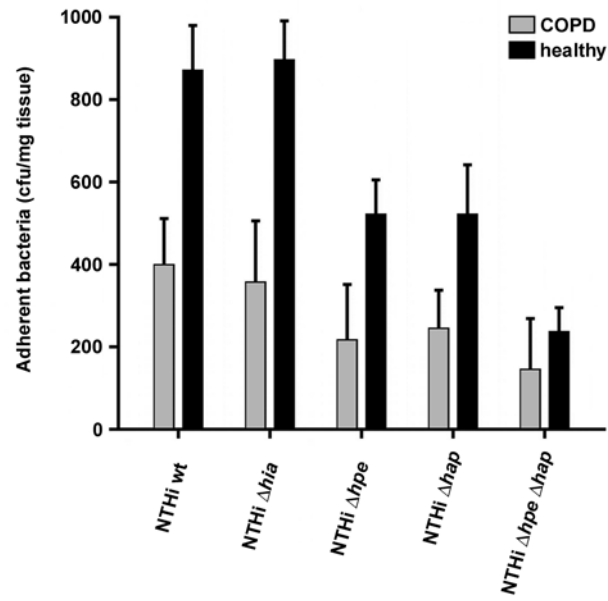

B

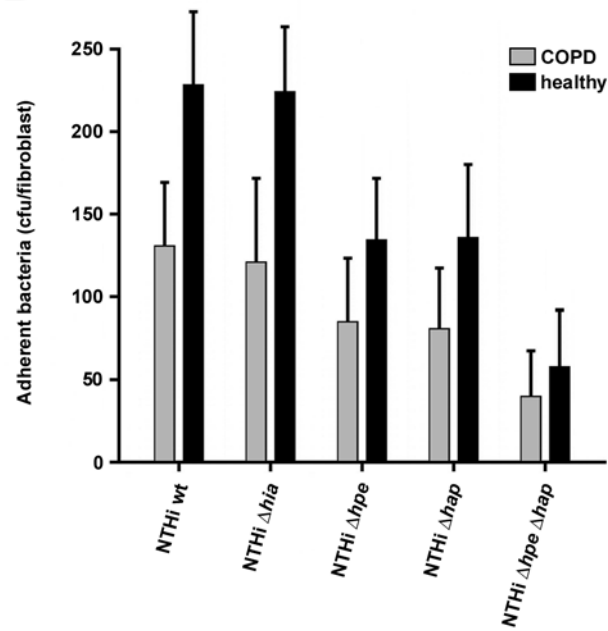

C

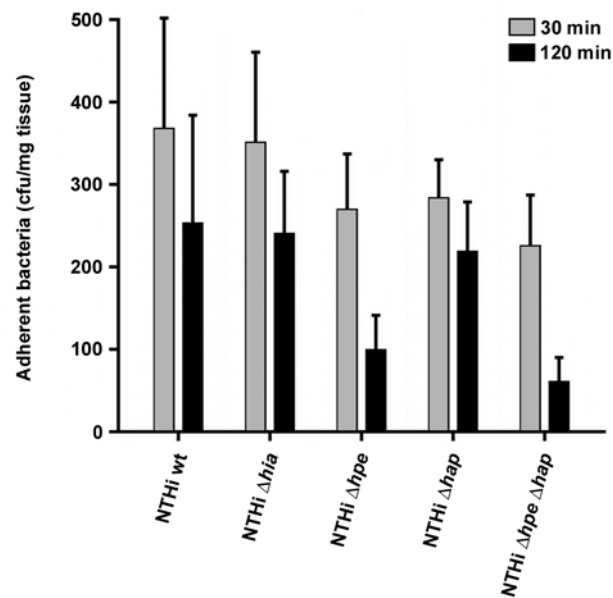

Supplement: Supplementary Figure S7 — Quantitative evaluation of bacterial killing in vivo. Tissue samples of murine control and COPD lungs inoculated with NTHi by intratracheal challenge were examined for the presence of bacteria by viable count assays (A). Similarly, the viability of NTHi bacteria was assessed in fibroblast adhesion cultures (B). (C), time-dependent killing of NTHi bacteria in vivo in inoculated mouse lungs as shown by viable count assays of homogenized lung tissue samples. [file Image_7.PDF]
